# Application of MALDI TOF and DART mass spectrometry as novel tools for classification of anaerobic gut fungi strains

**DOI:** 10.1007/s00216-025-05846-8

**Published:** 2025-03-25

**Authors:** Markus Neurauter, Julia M. Vinzelj, Sophia F. A. Strobl, Christoph Kappacher, Tobias Schlappack, Jovan Badzoka, Sabine M. Podmirseg, Christian W. Huck, Matthias Rainer

**Affiliations:** 1 Department of Microbiology, Universität Innsbruck, Technikerstraße 25d, 6020 Innsbruck, Austria; 2 CCB-Center for Chemistry and Biomedicine, Institute of Analytical Chemistry and Radiochemistry, Universität Innsbruck, Innrain 80-82, 6020 Innsbruck, Austria

**Keywords:** Neocallimastigomycota, On-target lysis, Alternative strain discrimination, Solvent free MS

## Abstract

Anaerobic gut fungi (AGF) have emerged as promising candidates for optimized biogas and biofuel production due to their unique repertoire of potent lignocellulose-degrading enzymes. However, identifying AGF strains through standard fungal DNA barcodes still poses challenges due to their distinct genomic features. This study explored the applicability of matrix-assisted laser desorption ionization time-of-flight mass spectrometry (MALDI) and direct analysis in real-time (DART) mass spectrometry (MS) as alternative methods for AGF identification. Further, the capability of the methods to differentiate strains from different growth phases was investigated. The study found that both MALDI and DART were viable methods for AGF strain identification. MALDI proved to be a precise and robust technique for strain discrimination with prediction accuracies of 94% for unknown standard samples. Even at longer growth times (>3 weeks) MALDI achieved good prediction accuracies with 84%; however, younger cultures (72 h) were only predicted with 63% accuracy. The fast on-target lysis with minimal chemical demand yielded suitable spectra for strain differentiation. DART MS, while effective with prediction accuracies of samples with the same age of up to 93%, exhibited lower prediction accuracies for cultures of different ages, with 14% for young (72 h) and 71% for old (>3 weeks) samples. Further research could enhance the capabilities of these mass spectrometry methods for AGF identification and broaden their application to species-level discrimination and a wider range of AGF genera.

**Graphical Abstract:**

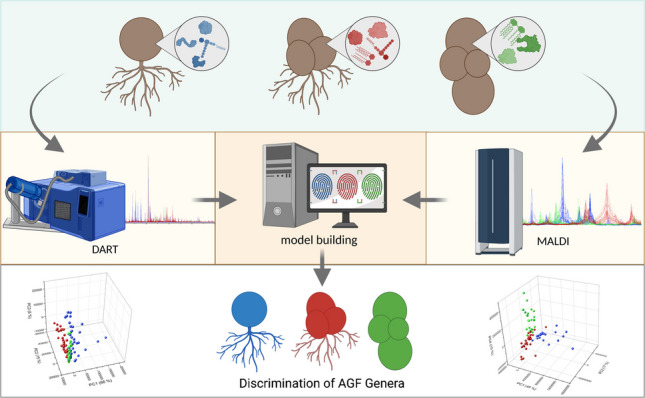

**Supplementary Information:**

The online version contains supplementary material available at 10.1007/s00216-025-05846-8.

## Introduction

Anaerobic gut fungi (AGF) are a phylum of strictly anaerobic fungi that inhabit the gastro-intestinal tract of herbivores. They encompass the basal fungal phylum of *Neocallimastigomycota* [1, 2]. AGF have been isolated from domesticated mammals, such as cattle, sheep, or goat, but also from wild mammals, such as capybara, giraffe, or elephants. More recently, they have been isolated from other groups of host-organisms, such as marsupials or reptiles (tortoises) [3–5]. They are promising candidates for the improvement of biogas and biofuel production processes, due to their potent carbohydrate-active enzymes (CAZymes), and can thus foster degradation of lignocellulosic biomass in anaerobic digestion systems [6–8]. Still, AGF remain poorly understood, mainly due to challenges in their isolation and cultivation, their sensitivity to oxygen exposure, and complex growth requirements, which limits the availability of (pure) cultures [6]. Mixed co-cultures with methanogenic archaea are more common, but more challenging to use for identification studies. There are currently 22 described genera of AGF, with the number steadily increasing over the last years [5, 9]. Culture-independent studies suggest the existence of more than double the current number of described AGF genera [10]. Recently, rank-assignment criteria for AGF have been recommended and the organization of AGF genera into four families has been proposed [11, 12].

The identification of AGF through classical DNA approaches can prove challenging as a result of their size heterogeneity within the universally used fungal ITS barcode region. Here, strain variation of up to 13% from clones of the same culture [13, 14] can be encountered. Hence, the AGF research community has moved towards more reliable marker gene regions (D1/D2 of the 28S rRNA gene region), but alternative identification methods are of high interest. Over recent decades, matrix-assisted laser desorption ionization time of flight mass spectrometry (MALDI) has been established as a rapid and reliable technique for the identification of strains in microbiological research. It has been shown to require less time than traditional microbiological approaches (e.g., DNA extraction and subsequent sequencing) and can be easily standardized using robust protocols. In this technique, the identification of strains is carried out through the spectral comparison, where certain marker peaks are present for each tested strain [15–17]. These usually arise from ribosomal proteins and are therefore highly useful for strain identification, as they are conserved housekeeping proteins with only small but significant variations among strains [18, 19]. While MALDI is well established for bacterial identification, identification of filamentous fungi is less routinely used [20]. Most studies on MALDI application focus on clinically relevant fungi and molds [21–24], and to our knowledge, no previous study has investigated the applicability of this method on AGF.

Over recent years, direct analysis in real-time mass spectrometry (DART) has also gained increased attention due to its ability to measure samples fast and with little pretreatment required. As opposed to MALDI, DART requires no extraction steps nor matrix co-crystallization, giving the power to directly measure samples [25]. While MALDI records spectra arising from high molecular weight proteins [15, 19], DART is employed in much lower mass ranges of usually 100 to 1000 m/z [25]. The applicability of this method for AGF identification could therefore yield additional benefits, as it would give a cheaper alternative and allow for characterization through a different mass range and hence different group of compounds. The two methods could therefore be established as alternatives or complementary methods.

This study aimed to investigate the feasibility of identifying AGF strains using MALDI. We employed on-target lysis to assess the effectiveness of this fast extraction method compared to more laborious methods with higher solvent demands. Further, the viability to discriminate strains at different growth stages was tested, to evaluate the robustness of the method for differentiation of cells of more heterogenous ages. Additionally, we investigated whether it was possible to identify AGF strains using DART without any extraction procedure. Again, the viability to discriminate strains of different growth stages was tested.

## Materials and methods

### AGF strain cultivation and conventional microscopic and DNA-based characterization

For this proof-of-concept study, three AGF pure cultures (i.e., without commonly encountered, associated methanogenic archaea) were tested for strain differentiation. They represented the available pure fungal cultures within the AGF culture collection at the Institute of Microbiology, Universität Innsbruck, and were chosen based on their different morphological characteristics and their taxonomic attribution to three distinct genera. Employed were as follows: *Anaeromyces mucronatus* (polycentric, filamentous growth; sequence accession number: ON614226 to ON614231), *Caecomyces communis* (monocentric, bulbous growth; sequence accession number: OP216660), and *Pecoramyces ruminantium* (monocentric, filamentous growth; sequence accession number: JN939127).

The strains were independently identified using light microscopy and through sequencing of the D1/D2 region of the LSU with the GGNL1F and GGNL4R primer pair [26]. The absence of syntrophic methanogenic archaea was confirmed by the absence of methane during GC measurement (GC-2010, Shimadzu) of the vial headspace, fluorescence microscopy, and PCR against the V4 region of the 16S-RNA using the 515f and 806r primer pair [27].

Fungal samples (dried fungal powder) were acquired as previously described in Neurauter et al. [28]. A total number of 80 samples was generated (*P. ruminantium*, *n* = 26; *C. communis*, *n* = 26; and *A. mucronatus*, *n* = 28). This set of samples is referred to as “core samples” or “1-week” throughout the text. To investigate discrimination at different growth ages, an additional sample set with “young” and “old” strains was produced. The young samples were grown for 72 h in 100 mL of growth medium [28], in order to ensure the production of sufficient biomass even after short growth times (*P. ruminantium*, *n* = 3; *C. communis*, *n* = 3; and *A. mucronatus*, *n* = 2). These samples are referred to as “72 h” throughout the text. The “old” samples were decaying cultures, harvested after cultivation for 3 weeks up to 3 months at 39 °C (*P. ruminantium*, *n* = 6; *C. communis*, *n* = 3, and *A. mucronatus*, *n* = 8). These samples will be referred to as “ > 3-week” throughout the text. Overall, a sample set of 104 fungal cultures was generated.

For MALDI a small amount of fungal powder (< 1 mg) was set aside for the on-plate extraction. For DART, the remaining powder was pressed to thin tablets with approximately 2 tons of pressure (Mini-Pellet Press, Specac Limited, Orpington, England).

### MALDI TOF measurements

MALDI TOF analysis was carried out with an Autoflex III smartbeam (Bruker AXS Inc., Madison, WI, USA) equipped with the flexControl software. As a matrix solution 0.5 mL α-cyano-4-hydroxycinnamic acid (CHCA) (Sigma-Aldrich) was prepared, by suspending 5 mg of CHCA in 250 µL acetonitrile, 237.5 µL water, and 12.5 µL TFA and vortexing the solution until the CHCA had fully dissolved. The on-target lysis was carried out according to Bader [29]: a small amount of dried fungal powder was deposited on the MALDI target plate. Then, 1 µL formic acid (70%) was added and dried for a few minutes. The spot was then overlaid with 1 µL CHCA matrix solution and again dried.

All spectra were acquired in the linear positive ion mode and a mass range of 1000 to 20,000 m/z. Spectra were generated with five profiles per spot and 1000 laser shots per profile. Ion sources were set to 20.06 kV (1) and 18.47 kV (2) and the lens was set to 7.32 kV.

### DART measurements

DART analysis was carried out using the ACQUITY QDa detector (Waters Corp., Milford, MA, USA) equipped with a DART SVP ion source (IonSense, Saugus, MA, USA). Helium was used as an ionization gas and mass spectra were recorded in a mass range of m/z 100–1000. For method optimization, the cone settings and temperature were varied in positive and negative ionization modes. The temperature was varied from 100 to 500 °C in 100 °C increments. For positive ion mode, 300 and 400 °C yielded equally good ionization. Hence, 350 °C was additionally tested and proved as the optimal temperature. For the cone settings, 10, 15, and 20 V were tried, with 15 V generating the most favorable results. As negative ion mode yielded inferior results, this approach will not be discussed in this manuscript.

The pressed tablets were held horizontally in close proximity to the entrance of the mass spectrometer. The DART ionization source was set up at an angle of 45° to yield higher ion counts. Each sample was measured three times for approximately 20 s and a mean of the spectra was created to give a representative spectrum of the sample.

### Data processing

MALDI spectra were initially processed using ClinProTools (Bruker Daltonics GmbH, Bremen, Germany). Spectra were baseline corrected (Top Hat) and recalibrated. Peak picking was carried out by two different approaches. First, manual selection of relevant peaks from the spectra of the core samples was done by choosing at least ten peaks with high in-strain homogeneity and high inter-strain heterogeneity. This manual selection was included done to improve comparability to the DART approach, where peaks had to be manually selected. For the second approach, a supervised neural network (SNN) was used to classify core sample spectra according to their reference strain, which also yielded relevant peaks for strain discrimination. The peaks/regions selected by the two approaches are found in the Supplementary Tables 1 and 2.

Multivariate analysis was then carried out in The Unscrambler X (CAMO Software) with both peak sets, generating principal component analysis (PCA). For the actual discrimination approach, a Mahalanobis discriminant analysis (MDA) was carried out. For the MDA, the dataset was randomly split with the common ratio of 70/30 into a calibration (56 samples) and a validation (24 samples) set. The calibration set was used to calculate the MDA to then predict the strains of the independent validation set. This was carried out in triplicate to ensure reproducibility from the random sample splitting. In order to assess the ability to identify strains with different ages, an MDA was calculated using the entire sample set of 80 core samples. With this MDA, the strains grown for 72 h and > 3 weeks were then predicted.

For DART, data analysis was carried out again with The Unscrambler X (CAMO Software). For the core samples, one *P. ruminantium* sample and, for the 72 h samples, one *C. communis* sample had to be excluded from the analysis, as not sufficient biomass was available to press a tablet. The analysis was carried out once with the full spectrum and once with selected marker peaks. The selected marker peaks were manually chosen through visual selection of relevant peaks in the spectra of the core samples, as described for the MALDI approach above. The selected peaks can be seen in Supplementary Table 3.

Again, PCA and MDA were carried out. For MDA, the sample set was randomly split at the ratio of 70/30 (positive: 55 calibration samples, 24 validation samples) to calculate the MDA from the calibration set and then predict the unknown samples of the validation set. This was again carried out in triplicate to ensure reproducibility from the random sample splitting.

The ability of the method to discriminate strains with different ages was assessed by calculating an MDA with the 79 core samples. This was used to predict strains grown for 72 h and > 3 weeks.

The generated files were deposited under 10.48323/fwvmz-nvd54. For this work, unused DART files in negative ion mode are stored there as well.

## Results and discussion

### MALDI

**Fig. 1:**
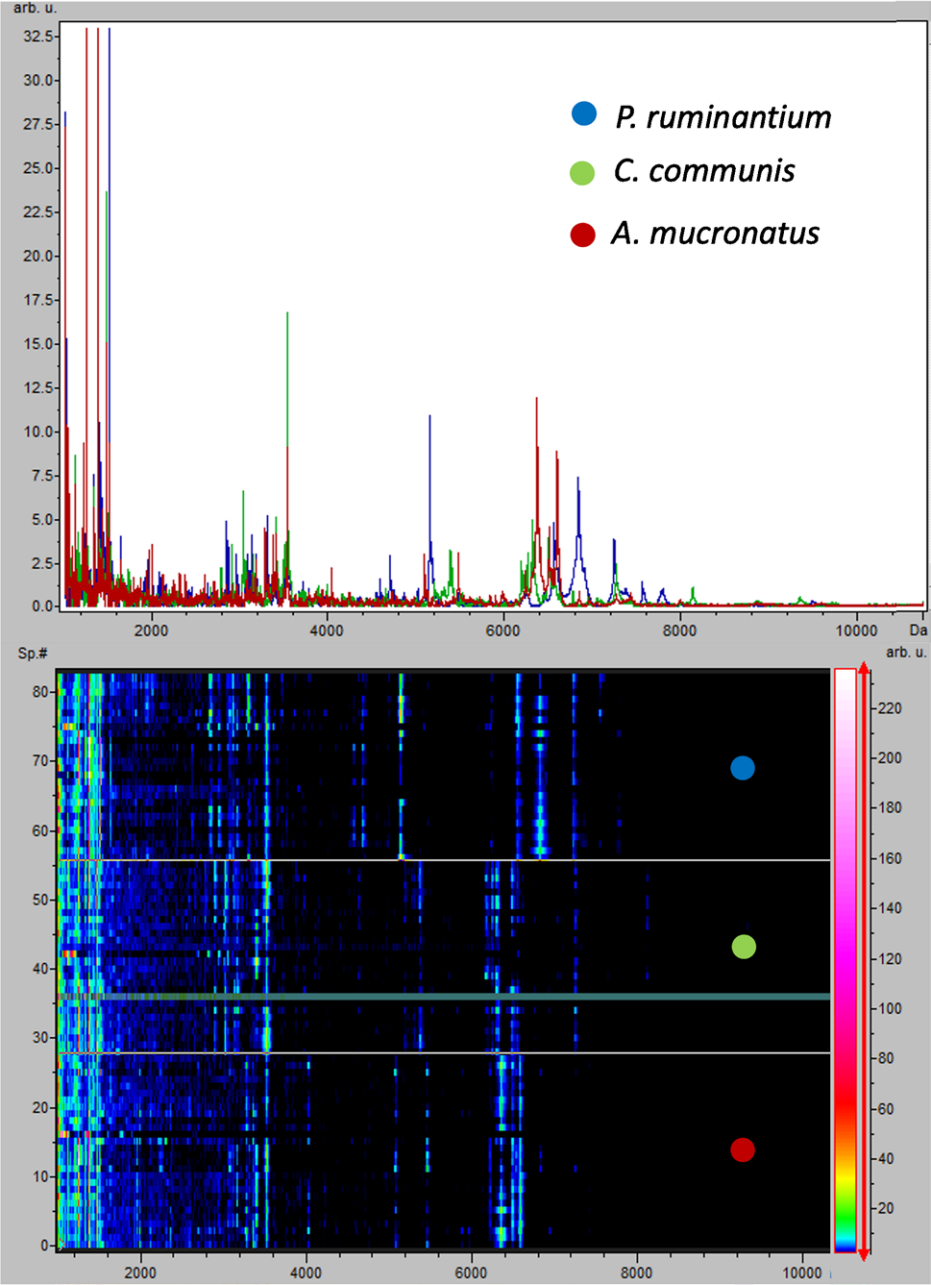
Average MALDI-TOF spectra of the respective AGF strains in the mass range of 1000–10,000 m/z shown as a line plot (top). In addition, all individual spectra of fungal samples are shown in a gel view (bottom) for easier identification of differences between the strains. The green line in the gel view represents a spectrum, for which recalibration for this format was not possible

For all strains tested, no relevant peaks over 10,000 m/z were detected (Fig. S1), which is why spectra are shown in mass range 1000–10,000 m/z (Fig. 1). Average spectra of the fungal strains showed clear differences in peak positions and heights, especially in the region of 4000–8000 m/z. For the analysis, all manually selected peaks were taken from this region. Most peaks chosen by the SNN approach were also found within this region that commonly includes the ribosomal subunit proteins of fungi [30].

**Fig. 2:**
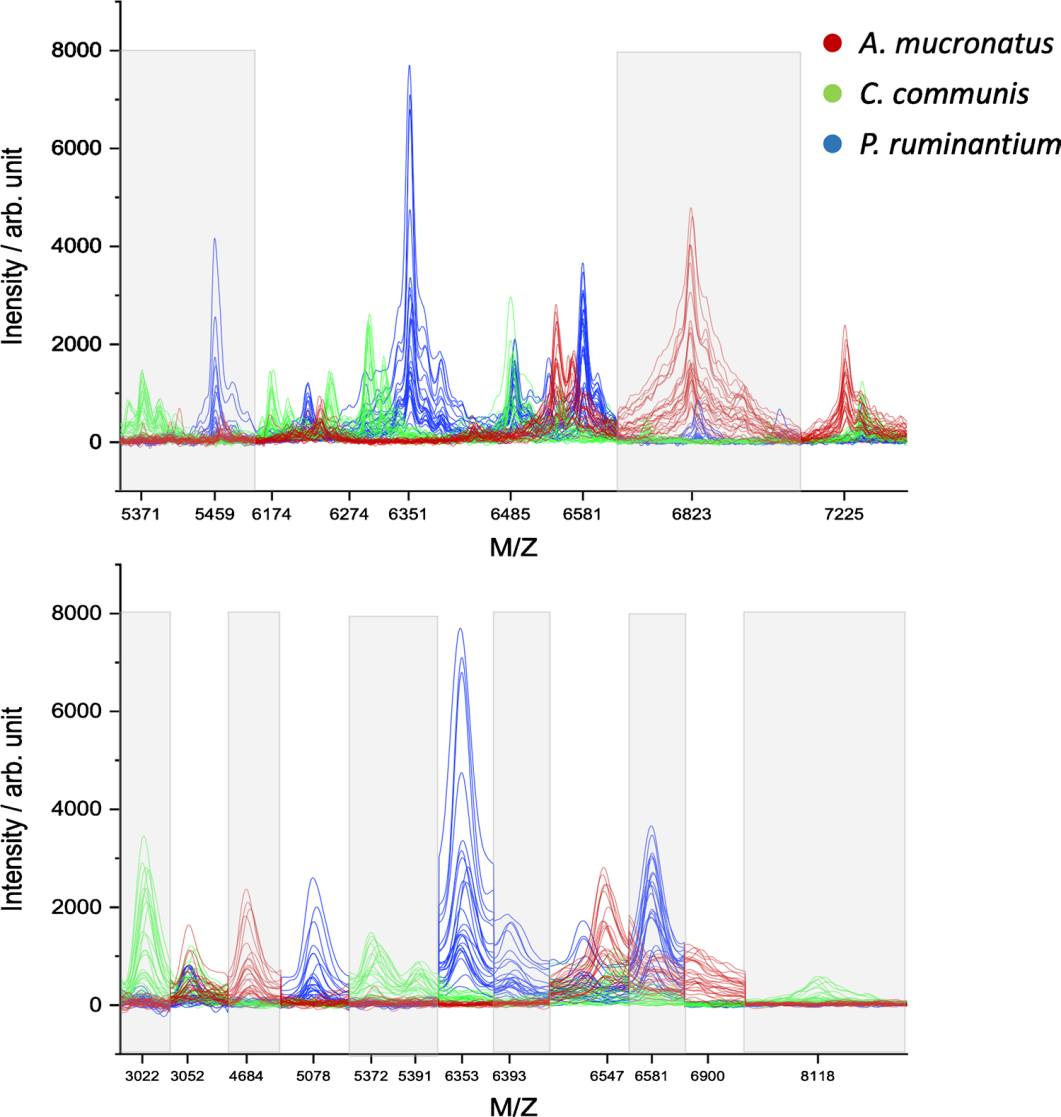
The selected peaks/regions from the MALDI-TOF spectra of fungal strains. Individual spectra are shown. Different selected segments are highlighted through shaded/non-shaded areas (specific regions given in Supplementary Tables 1 and 2). Top: Manually selected peaks/regions for strain discrimination. Bottom: Peaks/regions selected by the SNN for strain discrimination

Manual peak selection resulted in the selection of four regions, sometimes containing multiple peaks, that showed clear differences between the tested strains (Fig. 2). The gel view (Fig. 1) was used to pick regions with strongest differences among strains and low in-strain heterogeneity. Compared to the SNN, manual selection led to larger regions along the x-axis, including peak shoulders, being factored in, while the model in the SNN approach mostly chose individual, narrow peaks. The region around m/z 6900 exhibits peaks from *A. mucronatus* strains, but for some samples only peak shoulders are observed (Fig. 2), likely due to the interference of peaks from other strains in the nearby regions.

All three strains were clearly separated in the two PCAs with both the SNN and manual approach (Supplementary Fig. 2). For manually selected peaks, *A. mucronatus* showed larger intra-group heterogeneity, while for SNN selected peaks, *P. ruminatium* samples showed larger heterogeneity. With 82% for manually selected and 85% for SNN selected peaks, both PCAs displayed a large coverage of explained variance in the first three components. The clear separation in the PCA, which focuses on explaining largest variance, already foreshadows a satisfactory classification in the discriminant analysis, where inter-group separation is tried to be maximized [31].

For the manually selected peaks, the MDA model always had a model accuracy of 100%. The unknown validation set was predicted with an accuracy of 94% (± 2%). For the SNN approach, each of the models showed an accuracy of 100%. The unknown validation set was predicted with an accuracy of also 94% (± 2%).

The prediction accuracies for both models are very high, with small standard deviation. This renders the MALDI approach a precise as well as robust method for strain identification of AGF. In comparison, this is much higher than the probability of correct identification of 62% for ITS and 75% for LSU reported for early-diverging fungal lineages, which contain the *Neocallimastigomycota*, by a round-robin test for fungal DNA barcoding [32]. However, one has to consider the progress made with the design of specific primers for AGF detection and/or quantification in recent years. This likely leads to better results with state-of-the-art, DNA-based identification techniques, for which, however no current round-robin test study for success rate evaluation is available [33–35].

In addition to identification accuracy, it is important to consider other factors associated with standard nucleic acid-based techniques. For instance, Schoch et al. [17] found that the PCR amplification success rate for early-diverging fungal lineages was only 65%. The MALDI method, which does not rely on PCR amplification, offers a significant advantage for assigning AGF to reference spectra. Furthermore, this approach eliminates the need for chemicals in nucleic acid extraction and sequencing [36], aligning with the principles of green chemistry [37] and reducing potential biases introduced during nucleic acid extraction and PCR amplification.

For some studies with filamentous fungi, significantly lower prediction accuracies were observed when comparing the on-target lysis to full extraction methods in MALDI TOF analyses [38, 39]. Other studies, however, found high prediction accuracies for the on-target lysis method [40–42]. For AGF strains, the high prediction accuracy of the current study demonstrates the on-target lysis MALDI-TOF approach as a sufficient strategy for identification, requiring only minimal time and chemical consumption when compared to DNA extraction procedures [36] or even full extraction procedures for MALDI TOF identification [29, 41].

The SNN approach yielded the exact same results for strain classification through MDA construction as the manual selection of peaks for the core sample data. This again reinforces the robustness of the MALDI TOF method for fungal strain identification, showing that complex algorithms for peak selection might not be necessary.

**Fig. 3:**
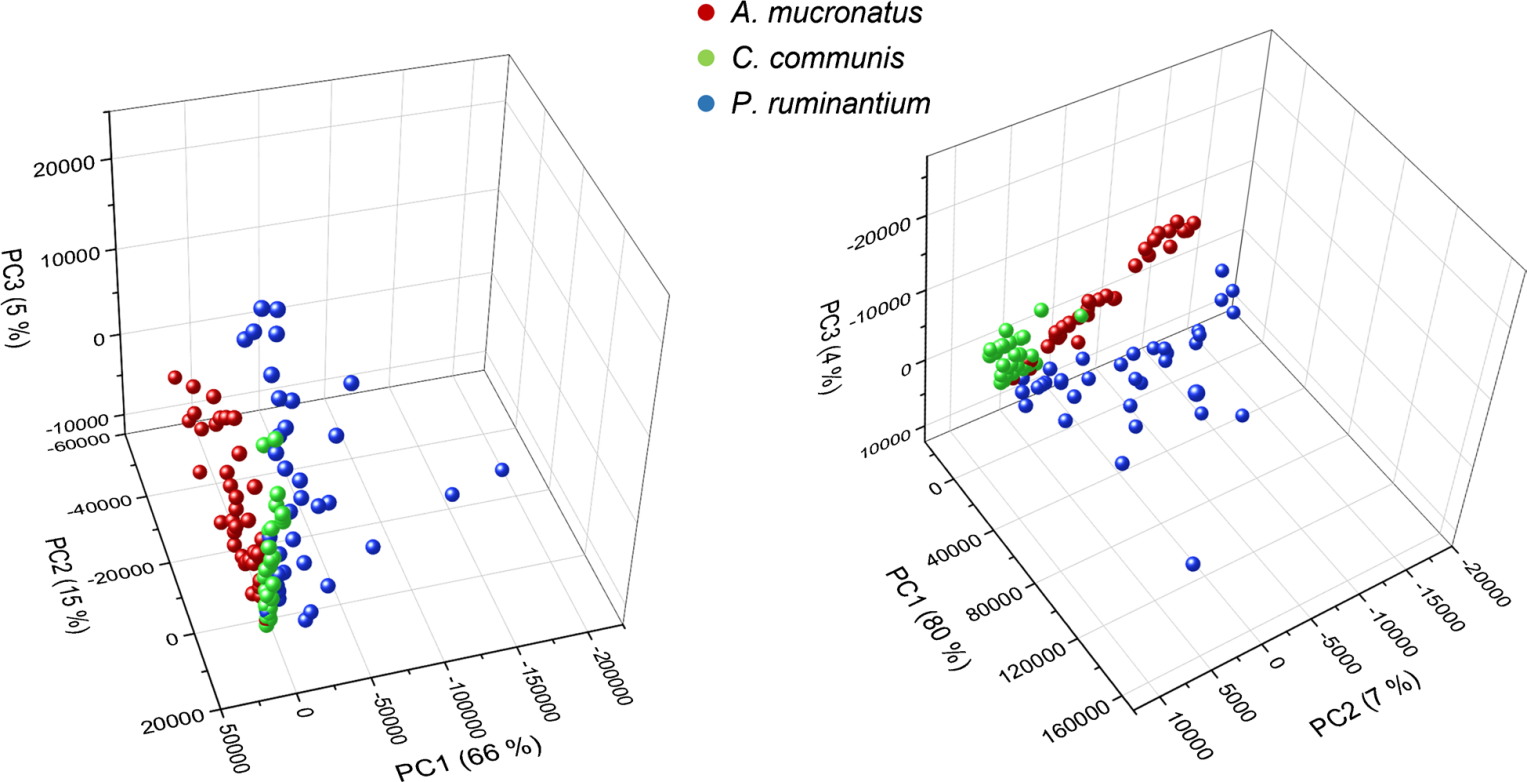
PCA of the MALDI TOF data of all samples, including core, 72-h, and > 3-week samples. Left: PCA using the manually selected peaks/regions. Right: PCA using the peaks/regions chosen by the SNN approach

When including the entire sample set (core, 72 h, and > 3-week samples), all three AGF strains still show relatively coherent clusters in the PCA graphs (Fig. 3). However, the separation is not as good when compared to the PCA of only core samples (Supplementary Fig. 2). For both approaches, visual peak selection and selection by SNN, the largest intra-group heterogeneity was observed for *P. ruminantium* samples. While PCA graphs for both approaches achieved a high coverage of explained variance when clustered by strain (86% and 91%, for manual selection and SNN, respectively), clustering by culture age led to no clear separation (Supplementary Fig. 3). This was true for both peak selections, which indicates the ability of the MALDI-TOF method to discriminate strains, even when cultures at different growth stages, harvested at different times, are used.

For the manually selected peaks, the MDA model of the core samples showed an accuracy of 94%, while for the SNN peaks it showed an accuracy of 96%. The prediction accuracies of the unknown 72-h and > 3-week samples by the respective MDA model can be found in Table 1.

**Table 1:** Prediction accuracies for 72-h and > 3-week samples based on MDA models derived from core sample spectra. For comparison, the original prediction accuracies of the core sample set (1 week) were also included

	Manual selection	SNN
72 h	50%	75%
> 3 weeks	88%	94%
1 week	94%	94%

The highest prediction accuracy was found for the SNN approach for > 3-week-old samples, equally high as for the prediction of the core samples. Manually selected peaks resulted in a slightly lower prediction accuracy for > 3-week samples. Both approaches had more difficulty classifying the 72-h samples, with the SNN this time reaching a much higher prediction accuracy over the manual selection. Issues with the identification of young fungal cultures have previously been reported [39, 43]. Both Packeu et al. [42] and Coulibaly et al. [46] found strongly increasing prediction accuracies when growth times of cultures were increased from 3 to 6 or 7 days for clinically relevant fungi and molds. The aging of fungal cultures corresponds to the production of secondary metabolites, pigments, and can also lead to differential abundances of marker proteins [40, 44]. Previous investigations with near-infrared spectroscopy have led to the conclusion that protein content of AGF increased over time [28]. This was also corroborated by Gay [45] who found increasing protein content in AGF with culture age. The emergence of higher protein contents in the AGF biomass over growth time could be a reason for the particularly challenging identification of 72-h samples, with insufficient amounts of marker proteins present at this time. Equally accurate identification of > 3-week-old cultures and core samples indicates that the protein signature obtained from fungi remains largely stable over longer time periods after an initial growth phase of > 72 h. This is in accordance with the belief that MALDI classification of strains relies on peaks originating from ribosomal proteins. As these are basic and have high proton affinity, they are often abundantly observed in MALDI spectra. This facilitates easier identification of strains even at various culture ages, as the housekeeping ribosomal proteins are plentifully present during all later growth stages. Further, differences in the weight of ribosomal proteins are a result of molecular evolution [19, 30]. The molecular masses of ribosomal proteins are therefore founded in phylogenetic differences of the underlying DNA sequence. These are more robust for strain differentiation as they do not change during developmental stages, such as the general proteome and metabolome would [30].

The higher prediction accuracies of the SNN selected regions indicate that for more complex identification approaches, as presented by the cultures of different ages, the peak selection by the algorithm is superior to visual peak selection. This could be caused by the sharper cutoff of the peaks chosen by the SNN approach, as compared to visual selection, where larger peak shoulder regions were included. In these regions, confounding new signals from other proteins that are produced throughout the fungal life cycle could be present for younger or older samples that complicate strain separation. Overall, SNN should therefore be preferred as the more robust method, allowing for better identification even after longer growth times.

#### DART

**Fig. 4:**
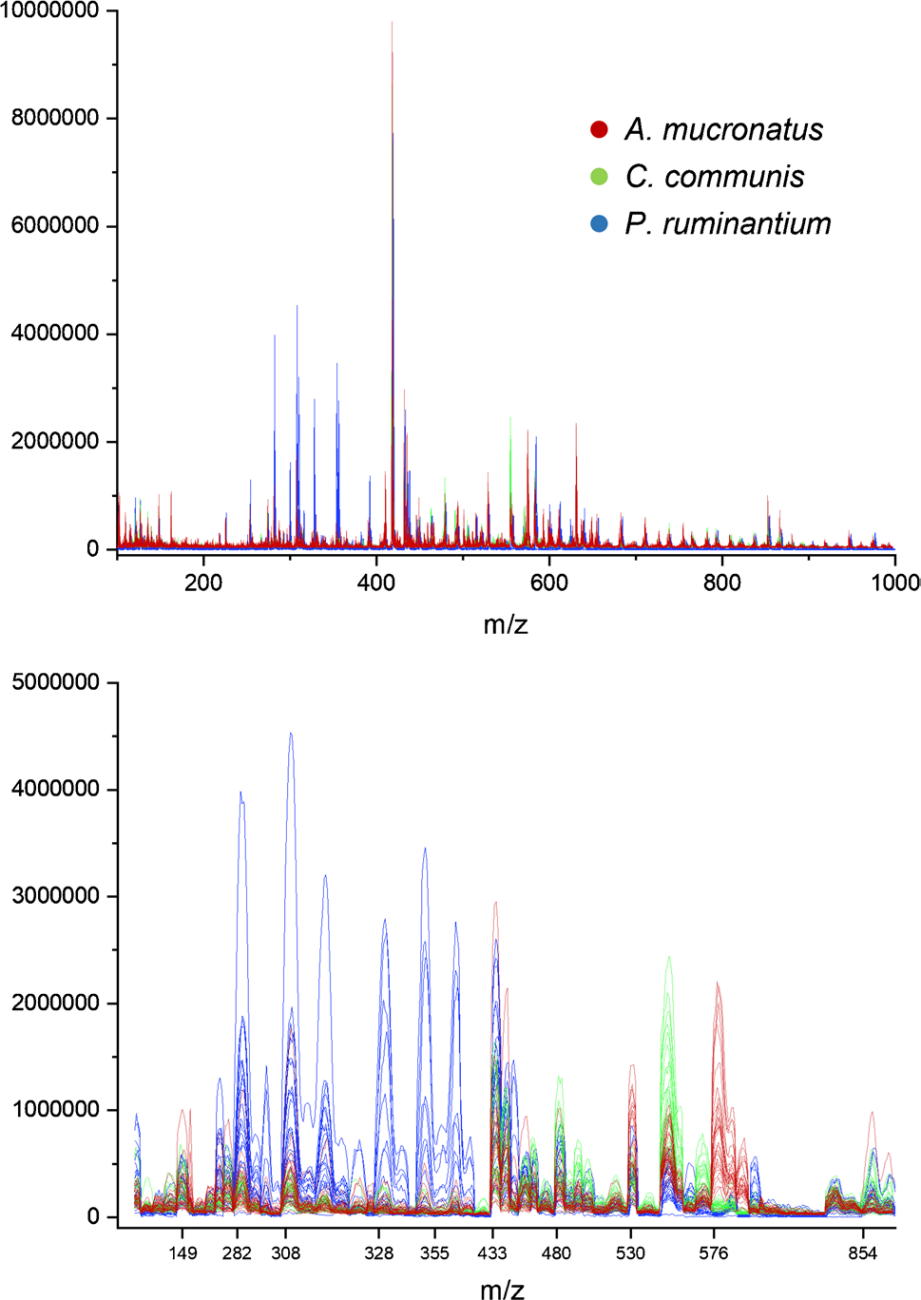
Individual DART spectra of the three fungal strains from the positive ion mode. Top: Complete spectra in the m/z range of 100–1000. Bottom: Manually selected peaks that showed clear differences between AGF strains. Details regarding the selected peaks can be found in Supplementary Table 3

The DART spectra revealed that the most prominent peaks appear between 250 and 700 m/z. *P. ruminantium* samples exhibited a region with multiple strong signals between 280 and 360 m/z (Fig. 4). This did not occur in other mass ranges for the other two strains. The spectra of manually selected peaks showed strong coherence of absorption within strains and large differences between strains.

**Fig. 5:**
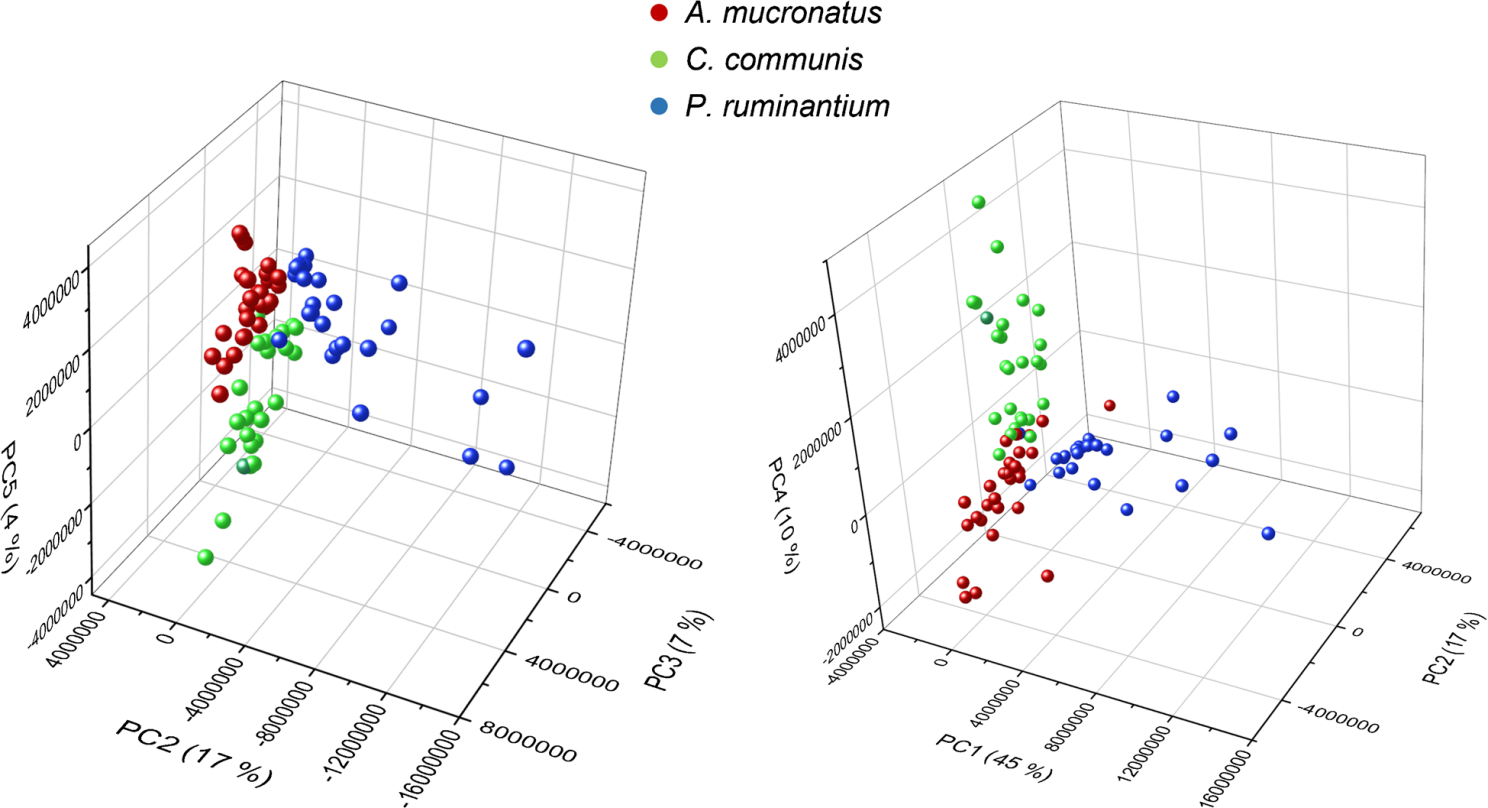
PCAs of core sample DART spectra in positive ion mode. Left: PCA using the full mass range. Right: PCA using the manually selected marker peaks

The PCA graphs of core sample DART spectra from positive ion mode showed clustering and separation of samples for both the full mass range and the selected marker signals (Fig. 5). However, to visualize adequate separation in the full mass range, PC2, 3, and 5 were used, resulting in an overall visualization of variance of merely 28%. This is likely caused by the influence of strong peaks, which show no coherence between signal intensity and the respective strain. The peak at m/z 419, for example, showed varying intensity for all samples among the three strains. This peak strongly influenced PC1, which explained 53% of variance, as can be seen in the loadings plot for the full range PCA (see Supplementary Fig. 4). However, PC1 did not aid in separation of the fungal samples and is therefore not shown in the PCA graph (Fig. 5). For the PCA using the manually selected marker peaks, PC1, 2, and 4 were used, resulting in higher explained overall variance of 72%. Peaks with irregular intensities, not corresponding to differences among strains, were removed in manual selection of the marker peaks, therefore resulting in a better explanation of variance when trying to separate the fungal clusters. For example, the peak at m/z 419 was removed in this set of peaks (Supplementary Table 3), leading to reduced influence from this signal in the PCA.

Using the full mass range, the MDA model calculated with the calibration sets exhibited an average model accuracy of 99% (± 1%). The unknown calibration set was predicted with an accuracy of 93% (± 2%). For the specific marker signals, model accuracy was 100% for each replicate. The unknown calibration set was again predicted with an accuracy of 93% (± 3%). This indicated that both approaches yield a high accuracy for prediction of unknown AGF samples, rendering the approach viable for AGF characterization.

No significant differences between MALDI and DART approaches in prediction accuracies of core samples were found through the Wilcoxon test (*p* = 0.1667). Both methods could therefore be employed for the identification of fungal strains at equal ages. Comparability between the methods is only feasible when using manually selected peaks, as both approaches yielded similar results for each technique. The benefits of the DART method over standard molecular approaches are similar to those previously described for MALDI, with the same limitations also applying here (see MALDI discussion). An additional benefit is the reduced chemical requirement, as DART eliminates the need for extraction or co-crystallization [25, 46]. Only minimal sample preparation is necessary (pressing of tablets). Additionally, lower acquisition costs of the DART instrument can be considered.

For the PCA graph including the full mass range, no satisfactory separation of the three fungal clusters was achieved (Supplementary Fig, 5). Only a discrimination of *P. ruminatium* samples from the other two AGF strains was possible. For the PCA graph based on marker signals, an acceptable classification and clustering of AGF samples were observed. However, results are not as clear as for the PCA using the core samples (Fig. 5). PCA graphs with color coding of culture ages using the full mass range revealed a partial separation of 72 h samples from older ones (Supplementary Fig. 6). Although the graph with marker signals does not show complete separation of the samples, it indicates some clustering of the individual AGF strains within the PCA. This separation and clustering within the PCA graph based on age rather than sample strain indicated that the MDA’s predictive performance for the DART approach is compromised when cultures of varying ages are used.

The MDA models based on core samples using the full mass range exhibited a model accuracy of 99%, while the model based on the marker signals showed an accuracy of 100%. The prediction accuracies of the models for the 72-h and > 3-week samples can be found in Table 2.

**Table 2:** Prediction accuracies for 72-h and > 3-week samples based on MDA models, derived from core sample spectra. For comparison, the original prediction accuracies of the 1-week old core samples were also included

	Full mass range	Manual selection
72 h	14%	14%
> 3 weeks	59%	82%
1 week	93%	93%

The prediction accuracies for 72-h and > 3-week samples for the DART method are much lower when compared to the MALDI approach. Here, a pattern of lower prediction accuracy for the 72 h samples is observed too. The apparent stronger coherence of mass spectra for 1-week-old and > 3-week-old samples can be gathered. The approach with selected marker signals for the core samples yielded a higher prediction accuracy for > 3-week-old samples. This could again be the result of no interference from strong signals, which do not correspond to differences among strains, in the marker signals. They rather seem to be an addition of noise to the data in the full mass range.

The mass range observed in DART focuses on much smaller molecules than the MALDI approach. The observed signals arise from smaller molecules, such as secondary metabolites or fragment ions of larger biomolecules [25, 47]. Their presence and relative abundance in the fungal biomass could vary much stronger during the growth and development of fungal cultures as compared to the large biomolecules, such as the ribosomal proteins, observed in MALDI. This could explain the considerably lower prediction accuracy of the DART approach for cultures of different ages.

### Comparison of MALDI and DART approaches

Both the DART and MALDI methods yielded high and robust classification accuracies for AGF strains when standardized growing conditions were employed. For MALDI, it has been demonstrated that the on-target lysis with minimal time and chemical demand was sufficient to yield practical spectra for strain differentiation. Additionally, SNN showed slight advantages over visual peak selection, and for DART, visual peak selection proved slightly better than using the full mass range. However, differences were only observed for classification of cultures at different ages, making the techniques robust enough for all peak selection processes when standardized protocols for fungal growth are used.

When cultures of different ages were used, MALDI outperformed DART in fungal classification. This is likely based on the different biomolecules being detected by the two methods. The ribosomal proteins found in MALDI spectra are not subjected to strong changes during fungal developmental phases [18, 19], while the presence and abundance of smaller molecules detected in DART can vary strongly over the growth of fungal cultures [25]. However, the classification of young fungal cultures (72 h) proved difficult for both approaches. This could hamper the application of these techniques when fast identification of strains is vital.

An additional benefit of the MALDI approach is the requirement of less biomass when compared to DART. For the DART approach, a substantial amount of freeze-dried fungal biomass was needed to press a tablet for analysis, while MALDI only required < 1 mg for extraction. This obstacle in DART could be avoided by also using transmission mode and application of sample-solvent suspensions on grids, which would then also only require similar sample amounts and solvents as MALDI. However, this would increase costs and chemical demand of the technique. Overall, MALDI seems to be a more robust approach for AGF classification, while DART has the benefit of being cheaper.

## Conclusion and outlook

This study shows a proof of concept for both the MALDI and DART approaches for AGF strain identification, even with its constraints of being limited to genus-level delineation and only including three out of the 22 AGF genera described. Both isolation and maintenance of AGF are laborious and expensive processes, and the absence of AGF in culture collections further limits the strains available to individual research facilities. We therefore encourage the inclusion of mass spectrometry data in the characterization of novel AGF isolates based on the procedures described in this study. Future work shall include more AGF strains, trying to achieve classification of more genera as well as differentiation of strains at, or even below, the species level. This would be especially interesting for monocentric, filamentous strains, which cannot be readily distinguished by light microscopy, and can even pose challenges to DNA marker-based identification. Moreover, AGF are commonly co-cultivated with syntrophic methanogenic archaea, which limits usage of these strains for mass spectrometry due to confounding signals from these methanogens. If corresponding archaeal MS libraries were to be constructed, these confounding signals could potentially be identified and removed, which could lead to the application of these techniques on AGF strains that are not yet available in pure culture.

Additionally, peak selection programs, such as SNN for MALDI, could be used for DART approaches to investigate the effect of such approaches on classification success. The robustness of the MALDI method due to the detection of mainly ribosomal proteins warrants investigation into identification of strains from more complex growth substrates, such as wheat straw or rumen extract, often used in AGF cultivation. An additional future outlook could be the inclusion of more strains of different culture age to further validate prediction accuracies of the constructed models. In the current approach, 72-h and > 3-week samples were predicted using an MDA model based on core sample data. With a higher number of young and old samples, a model including 72-h-old and > 3-week-old samples could be constructed. This would likely enhance the prediction abilities of the model for young and old fungal samples.

## Supplementary Information

Below is the link to the electronic supplementary material.

Supplementary file1 (DOCX 1658 KB)

## Data Availability

The generated MALDI and DART files for this work were deposited under 10.48323/fwvmz-nvd54. In this manuscript, unused DART files in negative ion mode are also deposited there.
