# Supplementary material for: Application of MALDI TOF and DART mass spectrometry as novel tools for classification of anaerobic gut fungi strains
Source: Anal Bioanal Chem. 2025 Mar 25;417(15):3245–56. doi: 10.1007/s00216-025-05846-8 (PMC12122643; doi:10.1007/s00216-025-05846-8)
Supplement: Supplementary file 1 — Supplementary file1 (DOCX 1658 KB) [file 216_2025_5846_MOESM1_ESM.docx]

**Application of MALDI TOF and DART Mass Spectrometry as Novel Tools for Classification of Anaerobic Gut Fungi Strains**

Markus Neurauter^1,2^, Julia M. Vinzelj^1^, Sophia F.A. Strobl^1^, Christoph Kappacher^2^, Tobias Schlappack^2^, Jovan Badzoka^2^, Sabine M. Podmirseg^1^, Christian W. Huck^2^, Matthias Rainer^2^

1 Universität Innsbruck, Department of Microbiology, Technikerstraße 25d, 6020 Innsbruck, Austria

* [m.neurauter@uibk.ac.at](mailto:m.neurauter@uibk.ac.at), +436606898295

2 Universität Innsbruck, Institute of Analytical Chemistry and Radiochemistry, CCB-Center for Chemistry and Biomedicine, Innrain 80-82, 6020 Innsbruck, Austria

Table S1: Peaks/regions selected manually from differences in the spectra in the MALDI TOF approach.

| **m/z of manually selected peaks/regions** |
| --- |
| 5346-5519 |
| 6164-6627 |
| 6722-6971 |
| 7164-7308 |

Table S2: Peaks/regions selected by the SNN model for differentiation of the strains in the MALDI TOF approach.

| **m/z of peaks/regions selected by the SNN approach** | | |
| --- | --- | --- |
| 3015-3030 | 5362-5401 | 6570-6597 |
| 3046-3064 | 6342-6367 | 6888-6918 |
| 4676-4698 | 6385-6411 | 8080-8167 |
| 5064-5093 | 6519-6558 |  |

Table S3: Manually selected marker peaks for DART positive ion mode.

| **m/z of selected peaks for positive mode** | | |
| --- | --- | --- |
| 121 | 326 | 507 |
| 126 | 329 | 508 |
| 130 | 344 | 531 |
| 140 | 354-358 | 536 |
| 144 | 386 | 556-558 |
| 163 | 433 | 571 |
| 1769 | 436 | 573 |
| 219 | 438 | 577-578 |
| 254 | 450 | 594 |
| 275 | 464 | 624 |
| 282-283 | 478 | 758-761 |
| 301 | 480 | 795-796 |
| 308-311 | 582 | 855-857 |
| 316 | 492 |  |


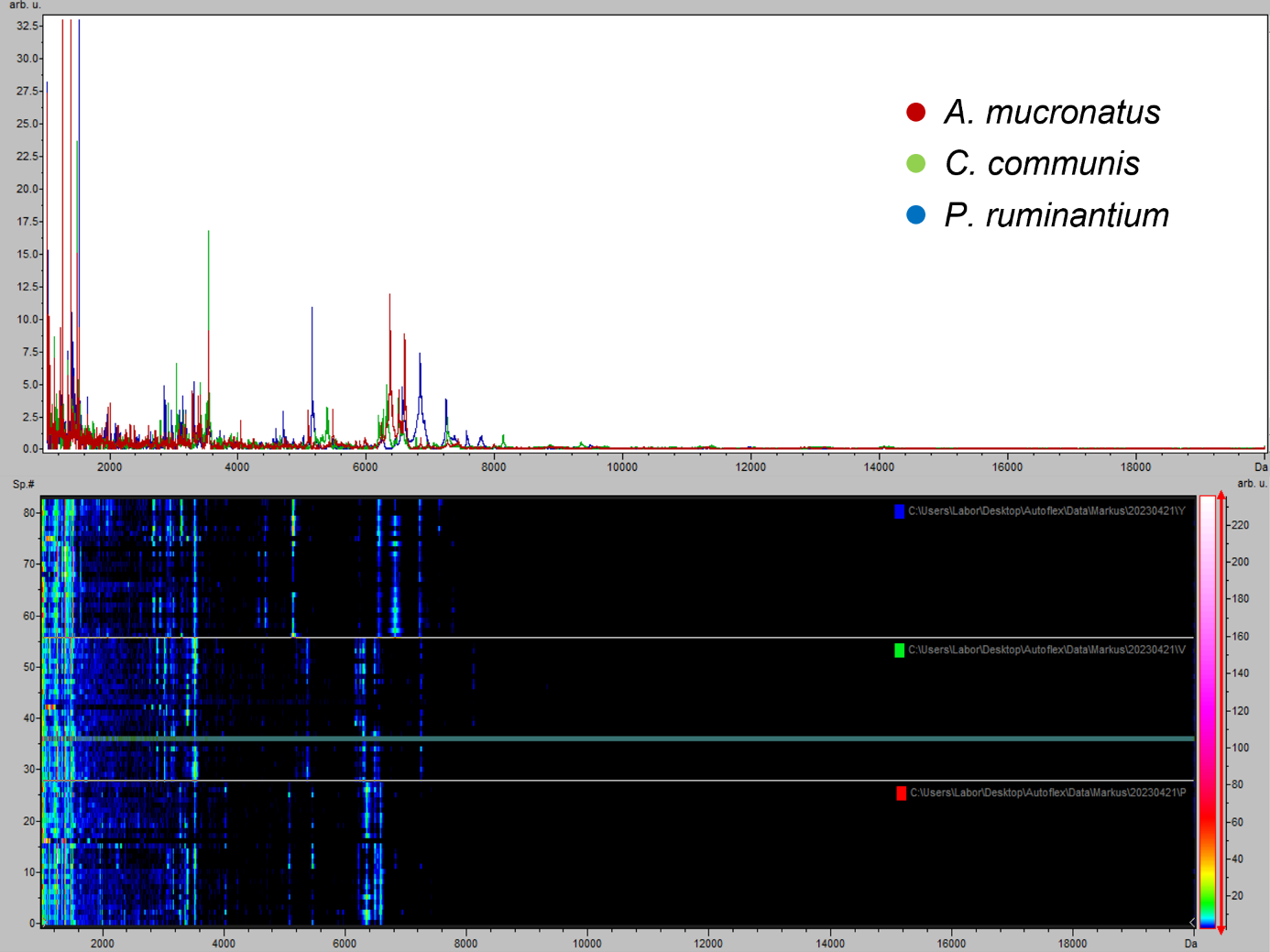


Figure S1: Average MALDI-TOF spectra of the respective AGF strains in the mass range of 1 000 - 20 000 m/z shown as a line plot (top). In addition, all individual spectra of fungal samples are shown in a gel view (bottom) for easier identification of differences between the strains. The green line in the gel view represents a spectrum, for which recalibration for this format was not possible


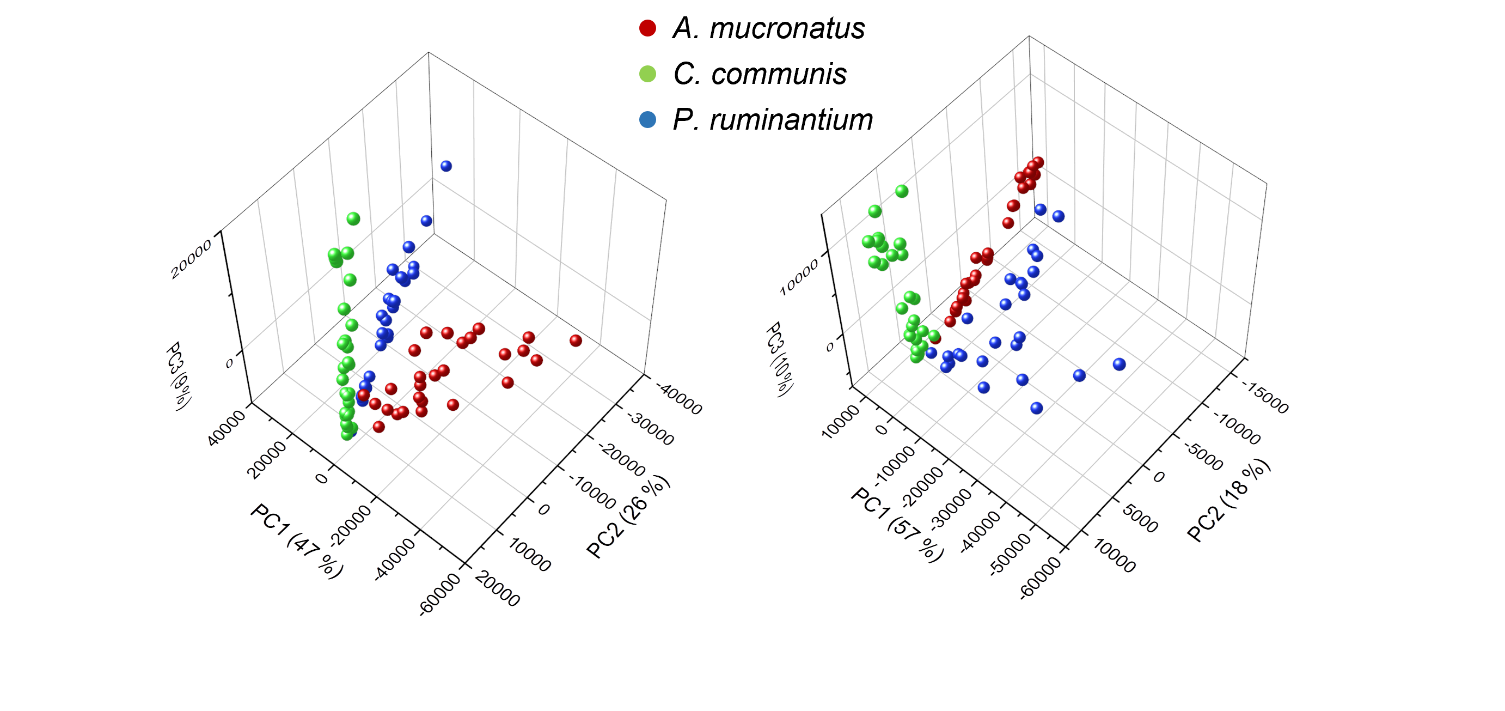


Figure S2: PCA of MALDI-TOF data for the core samples. Left: PCA using the manually selected peaks/regions. Right: PCA using the peaks/regions chosen by the SNN approach.


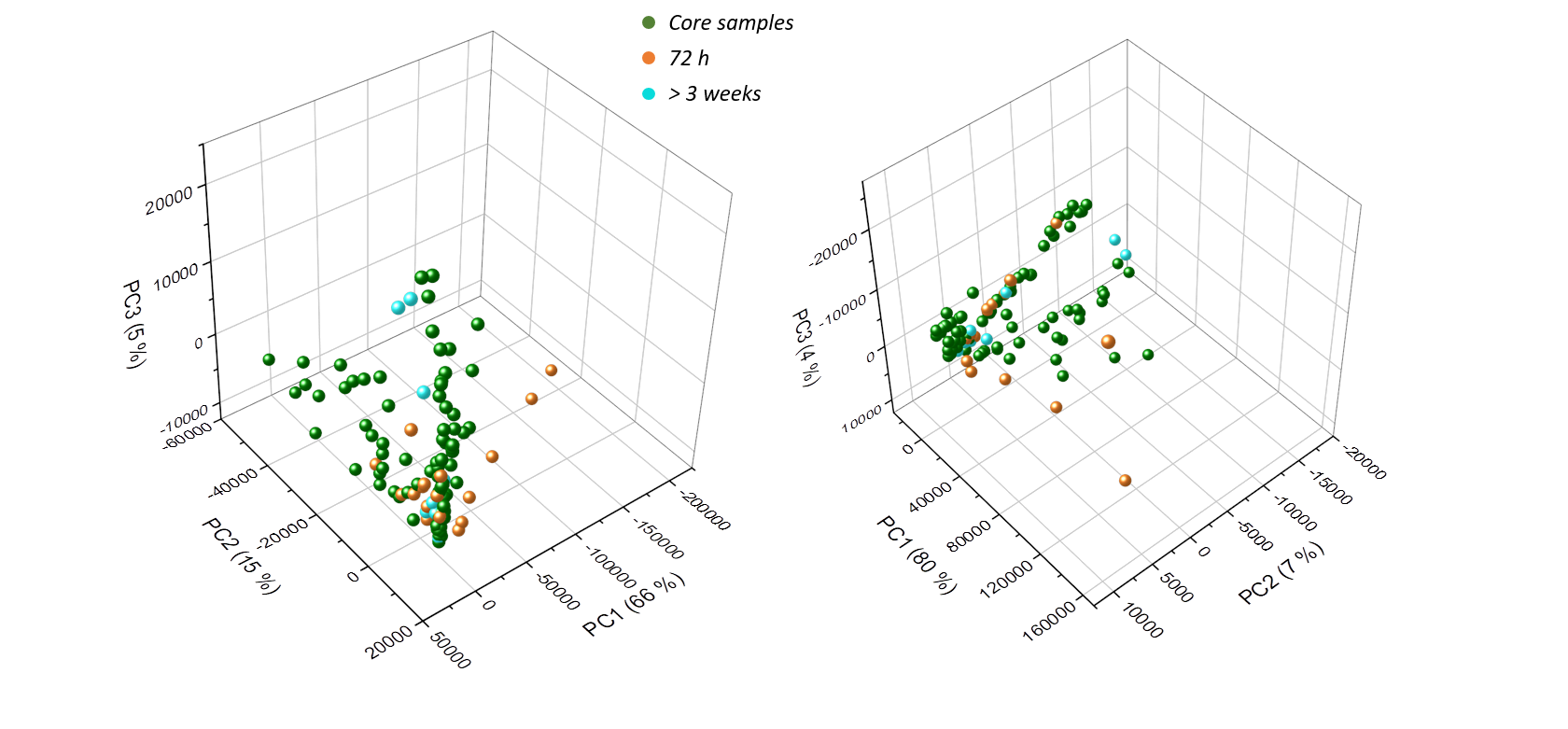


Figure S3: PCA of the MALDI-TOF data of all samples, including core, 72 h and > 3 week samples. Left: PCA using the manually selected peaks/regions. Right: PCA using the peaks/regions chosen by the SNN approach. The same depiction as in Supplementary Figure 1, but with color coding corresponding to the culture age.


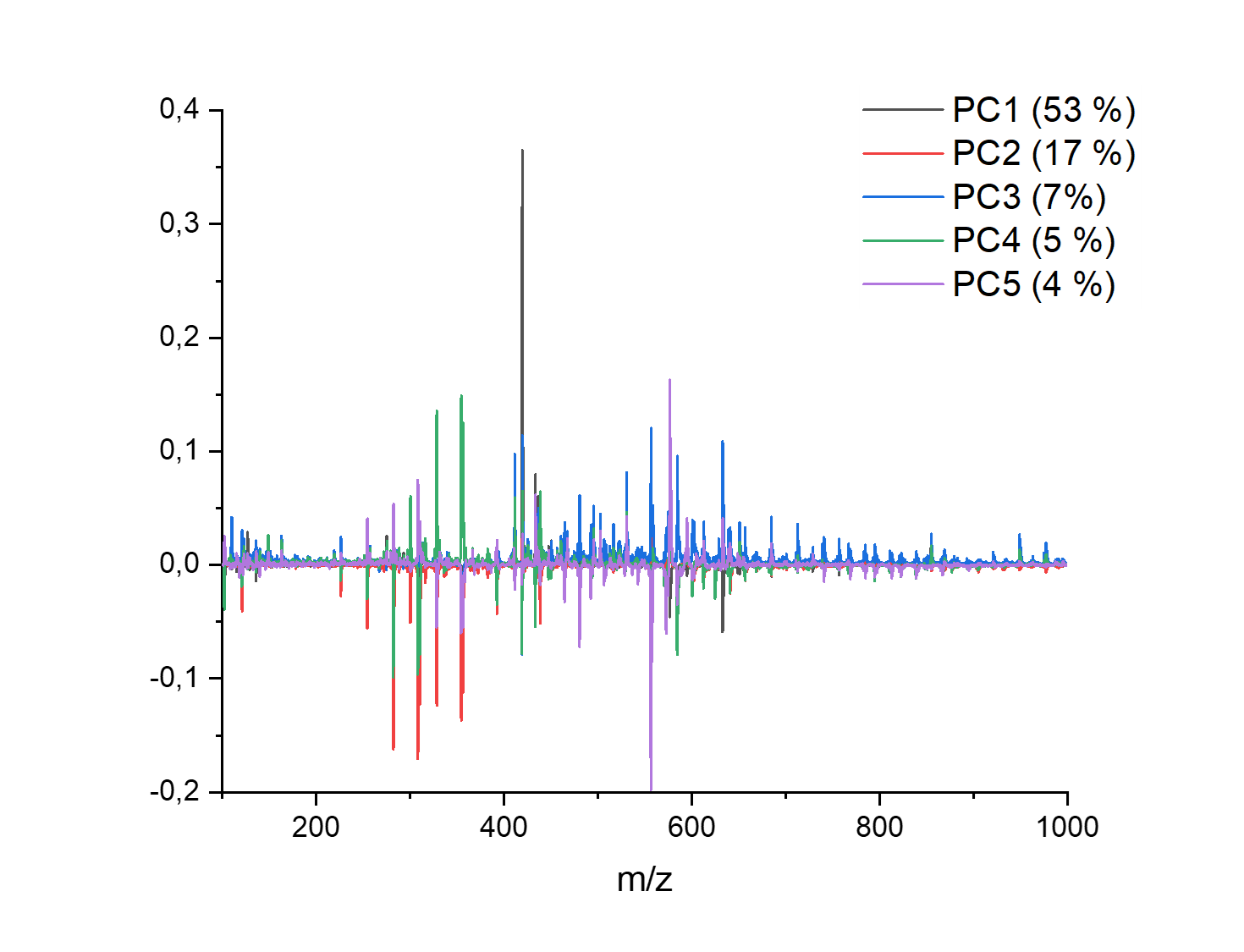


Figure S4: Loadings plot for the PCA of core samples in DART positive ion mode, using the full mass range of m/z 100-1000 Da.


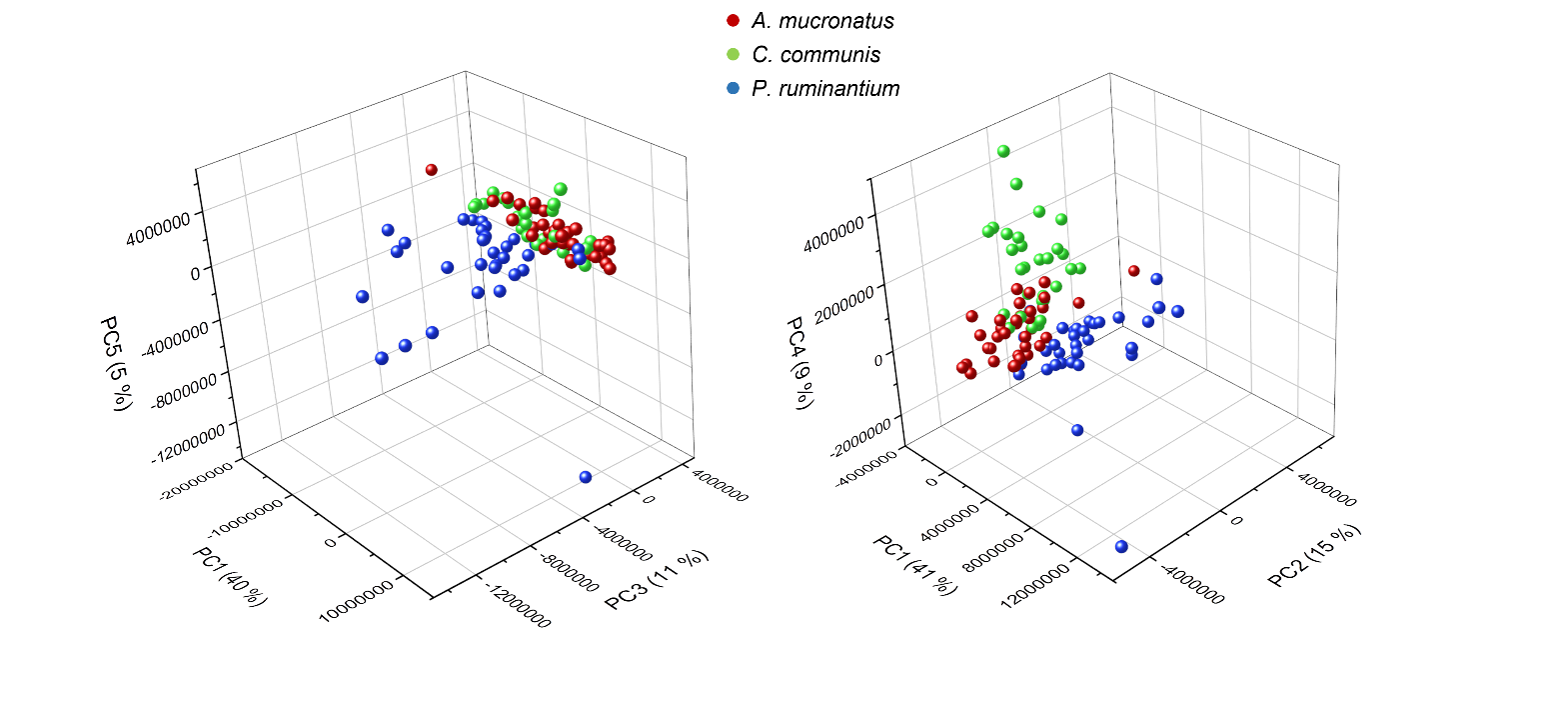


Figure S5: PCA of the DART data of all samples, including core, 72 h and > 3 week samples, measured in positive ion mode. Left: PCA using the full mass range. Right: PCA using the selected marker peaks.


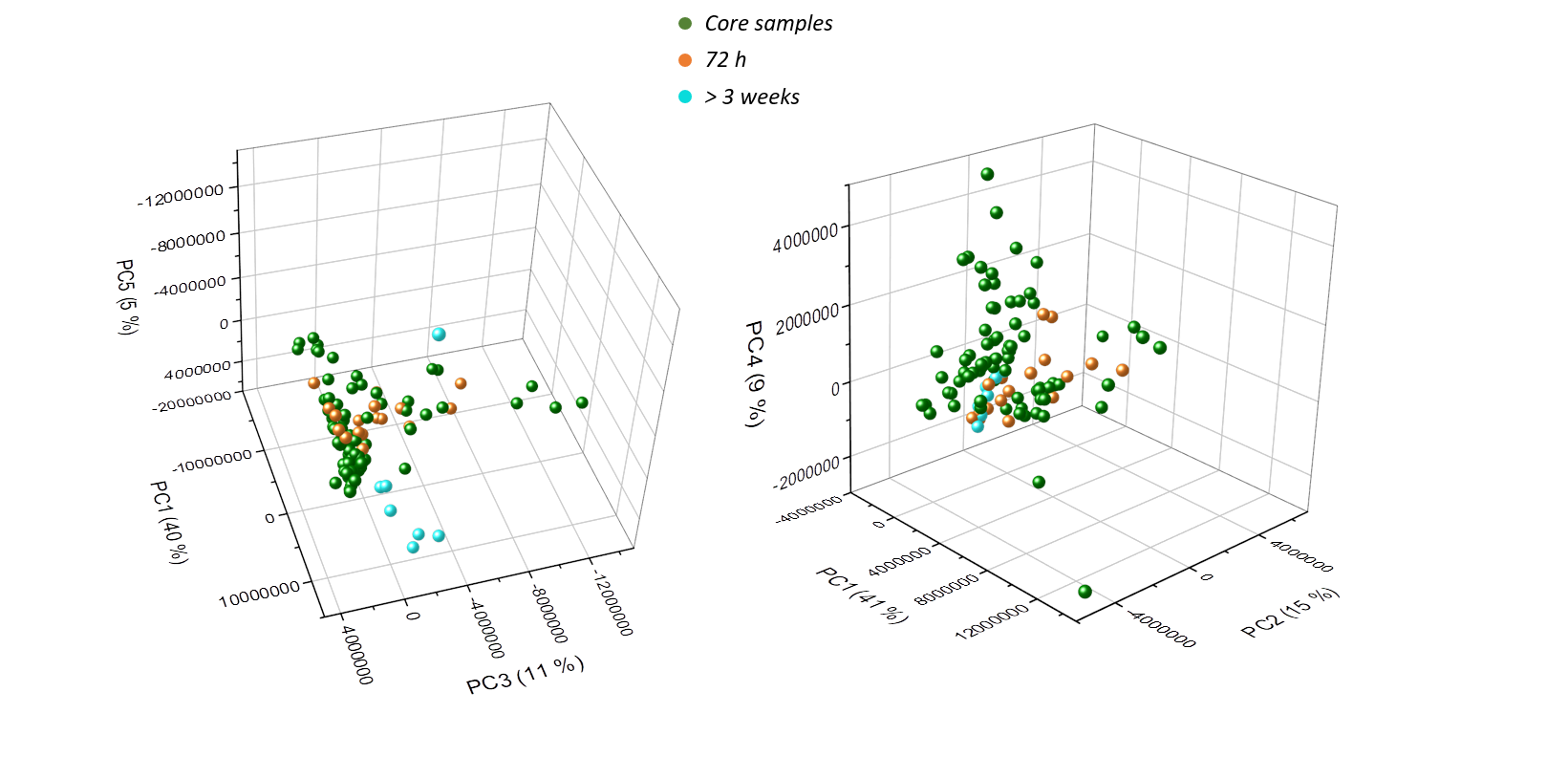


Figure S6: PCA of the DART data of all samples, including core, 72 h and > 3 week samples, measured in positive ion mode. Left: PCA using the full mass range. Right: PCA using the selected marker peaks. The same depiction as in Supplementary Figure 4, but with color coding corresponding to the culture age.
